# Acral melanoma: considerations about the surgical management of this tumor^☆^^☆☆^

**DOI:** 10.1016/j.abd.2019.09.019

**Published:** 2019-09-30

**Authors:** Lívia Mesquita Zyman, José Antônio Jabur da Cunha, Andrea Ortega Gimenez, Marcus Maia

**Affiliations:** Clinic of Dermatology, Department of Medicine, Santa Casa de Misericórdia de São Paulo, São Paulo, SP, Brazil

Dear Editor,

Acral melanoma (AM) is a subtype of cutaneous melanoma, which is found on the soles of the feet, palms of the hands, and subungual areas.^1^, ^2^, ^3^ It presents a lentiginous growth pattern and is more frequent in darker-skinned populations, including Africans, Asians, and Latin Americans. Despite its indolent behavior, AM has a poor prognosis, often because it is diagnosed at a more advanced stage, which makes dermatological training about this tumor very important.^1^, ^2^, ^3^, ^4^, ^5^ The treatment of AM is based on surgical removal of the tumor. The excision challenges the surgeon, since it frequently results in large surgical defects that are difficult to reconstruct. The best surgical technique for the affected areas should achieve good functional and cosmetic results, with a short healing time and a low rate of complications.

We present two cases that illustrate what we have observed in a reference center in São Paulo. A female patient, 49 years old, had AM excised and reconstructed with full-thickness skin graft (FTSG). The patient evolved with partial loss of the graft and intense local hyperchromia all over the graft attachment site (Fig. 1). The second case refers to a male patient, 60 years old, diagnosed with AM. Secondary intention healing (SIH) was preferred after excision. There were no complications, with a complete cosmetic and functional healing 12 weeks after surgery (Figure 2, Figure 3).

**Figure 1:**
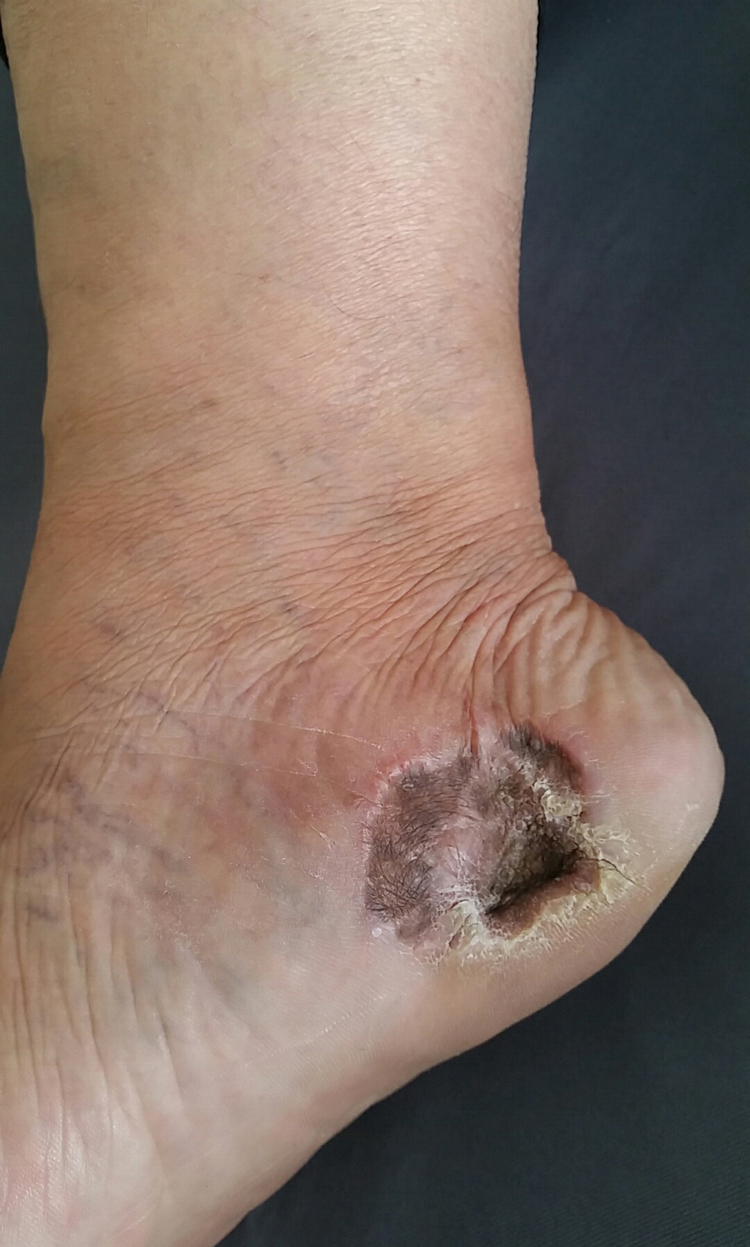
Final result after full thickness skin graft. Note the partial loss of the full thickness skin graft and the intense local hyperchromia all over the graft attachment site.

**Figure 2:**
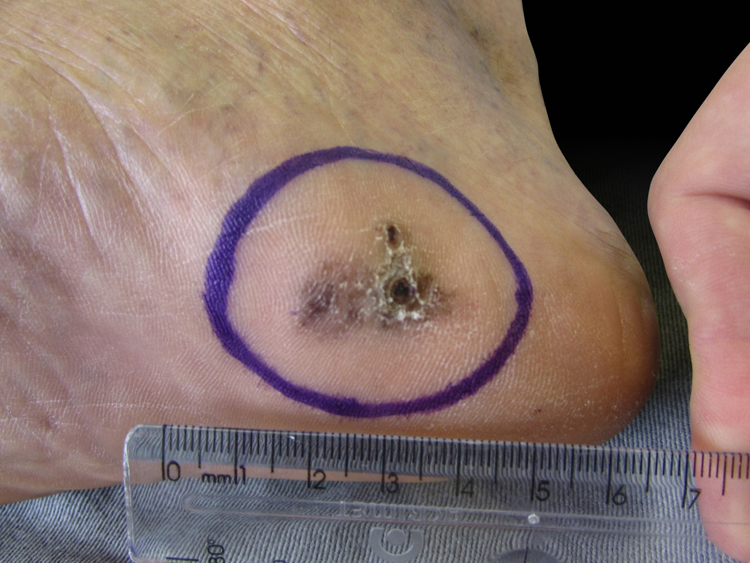
Preoperative aspect. Initial aspect of acral melanoma, excised with secondary intention healing.

**Figure 3:**
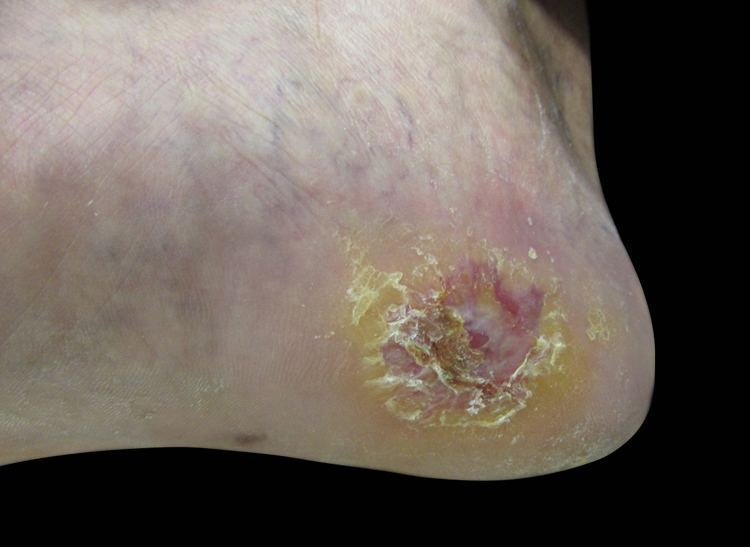
Final result after secondary intention healing. Photo taken after 12 weeks of follow up.

Clinically, AM appears as macules or nodules in the acral skin, and may present variations in color from brown to black and irregular borders. On the other hand, amelanotic lesions present pinkish-red macules or nodules that are often difficult to diagnose. Similar to other melanoma subtypes, their prognosis is determined mainly by their Breslow thickness at diagnosis, and then a wide surgical excision should be performed with an appropriate margin, including the subcutaneous fat. After surgery, the wound can be repaired using several methods, such as primary closure, SIH, local and free flaps, and FTSG.^1^, ^4^ In addition, the exact location of the lesion, comorbidities, age, and lifestyle of the patient should all be considered when repairing foot lesions.^1^, ^2^

Surgical defects on soles of the feet are difficult to repair due to the extreme lack of tissue mobility in this area. Besides, due to the characteristic lentiginous growth of the tumor associated with the safety margin, surgical defects following AM surgeries can rarely be repaired with primary closure, thus requiring the use of more complex techniques.^1^, ^4^

Although skin grafts have been frequently used for acral reconstructions, intense blackening during the healing process is frequent, which makes the use of such a technique inadequate not only for esthetic reasons but also for clinical follow-up when considering the possibility of local tumor recurrence. In addition, the plantar location impairs the nutrition of the grafts, which often evolve with partial or even total necrosis.

SIH has been considered a more effective method of repairing when compared to FTSG, although it requires a longer healing time.^2^, ^4^ It is indicated for areas more susceptible to pressure since the local inherent trauma of these sites tends to impair the viability of grafts and flaps.^4^ We can also point excellent cosmetic results since they do not evolve with blackening, which facilitates the clinical follow-up of these patients. Also, since it does not require donor area excision, SIH does not leave additional scars, as opposed to skin grafts and flaps.^1^, ^2^ However, the disadvantage is that SIH requires longer healing time, with more medical visits.

Jung et al.^2^ demonstrated that patients in the SIH group showed better results than patients submitted to skin graft repair when considering the occurrence of infections, seroma, and necrosis.^2^ In our experience, SIH has been a method that is practical, low-cost, and unlikely to present infectious complications.

In this letter, we presented two examples of possible surgical approaches for AM management, showing that, despite its longer recovery, SIH produces excellent cosmetic and functional results,^2^ with minimal morbidity and lower complication rates.^1^ Such findings encouraged us to initiate a prospective study to evaluate a larger number of patients with clinical outcomes similar to those reported in this study. In conclusion, our findings suggest that SIH approach has a potential role in the surgical treatment of the AM, which should be considered by surgeons.

## Financial support

None declared.

## Author's contributions

Lívia Mesquita Zyman: Approval of the final version of the manuscript; elaboration and writing of the manuscript; critical review of the literature; critical review of the manuscript.

José Antônio Jabur da Cunha: Approval of the final version of the manuscript; conception and planning of the study; obtaining, analyzing and interpreting the data; effective participation in research orientation; intellectual participation in propaedeutic and/or therapeutic conduct of the cases studied; critical review of the manuscript.

Andrea Ortega Gimenez: Elaboration and writing of the manuscript.

Marcus Maia: Obtaining, analyzing and interpreting the data; critical review of the manuscript.

## Conflicts of interest

None declared.
